# Research on the Impact of Marketing Strategy on Consumers’ Impulsive Purchase Behavior in Livestreaming E-commerce

**DOI:** 10.3389/fpsyg.2022.905531

**Published:** 2022-06-16

**Authors:** Bing Chen, Lei Wang, Hassan Rasool, Jun Wang

**Affiliations:** ^1^School of Foreign Languages for Business, Guangxi University of Finance and Economics, Nanning, China; ^2^School of Business, Guilin University of Electronic Technology, Guilin, China; ^3^School of Economics, Pakistan Institute of Development Economics, Islamabad, Pakistan

**Keywords:** e-commerce live streaming shopping, impulsive purchase behavior, People-Product-Place, marketing strategy, COVID-19

## Abstract

Livestreaming e-commerce has emerged as a highly profitable e-commerce that has revolutionized the retail industry, especially during the COVID-19 pandemic. However, research on livestreaming e-commerce is still in its infancy. This study sheds new light on impulsive purchase behavior in livestreaming e-commerce. Based on stimulus-organism-response (SOR) theory, this study introduces the “People-Product-Place” marketing strategy for livestreaming e-commerce from the perspective of consumer perception and aims to understand the impact of marketing strategy on impulsive purchase behavior in e-commerce livestreaming shopping scenes, and to examine the mediating effect of involvement. The study conducted SEM analysis, in Amos, on 437 response sets from an online anonymous survey. The results show that *perceived* e-*commerce anchor attributes*, *perceived scarcity*, and *immersion* positively influence impulsive purchase behavior; that “People-Product-Place” marketing strategy is important; and that effective marketing triggers impulsive purchase. *Perceived e-commerce anchor attributes, perceived scarcity*, and *immersion* positively influence *involvement*, which positively influences impulsive purchase. *Involvement* mediates between *perceived* e-*commerce anchor attributes*, *perceived scarcity* and *immersion*, and impulsive purchase. These findings guide marketers to improve the profitability of livestreaming e-commerce and provide some references of economic recovery for many other countries that also suffered from the impact of the COVID-19 pandemic.

## Introduction

New consumption patterns derived from the Internet, network, and information systems technology have emerged in recent years (Corcoran and Andrae, 2013). These developments have led to changes in individuals’ concepts of consumption and their consumption habits. After the web portal era of Web 1.0 and the social media era of Web 2.0, we have entered the scene media era of Web 3.0 (Zhang, 2020). The emergence of online shopping has greatly improved consumers’ shopping experience (Helm et al., 2020). Online shoppers are not restricted by time, location, or travel/transportation. However, in “traditional” online shopping, shoppers receive information only through images, text, and prerecorded video (Wongkitrungrueng and Assarut, 2020). Thus, in the Web 3.0 era the development of this e-commerce has entered a bottleneck (Wu Q. et al., 2020). Intentionally designed promotional videos and excessively beautified images of online products make it difficult for consumers to obtain true information. This “asymmetry of information” between online consumers and merchants contributes to consumer doubt and distrust in purchase decision-making (Demaj and Manjani, 2020; Lamr and Dostál, 2022; Utz et al., 2022). Lagging customer consultation services further frustrate the online shopping experience (Othman et al., 2020). Thus, innovation that emphasizes a comprehensively good consumer experience is needed.

China is the largest Internet market in the world (Akram et al., 2018a). In 2016, a new online retailing model integrating e-commerce with online livestreaming shopping emerged in China (Rui and Kang, 2016). This marketing model is based on e-commerce, uses livestreaming as a marketing tool (Ding et al., 2020), and provides direct and efficient communication to minimize information asymmetry (Wongkitrungrueng et al., 2020). This enables online shoppers to get a real three-dimensional experience in the virtual network environment and increases adhesion and trust between users, merchants, and platforms (Li, 2020). Professional selection of products, anchor persona, live product display, and real-time interaction is integrated into a retailing model that attracts users to watch, interact, and purchase (Liu L. et al., 2020). The COVID-19 pandemic has driven rapid change in product consumption patterns (Liu L. et al., 2020; Zwanka and Buff, 2021). Since 2020, the “home economy” trend has further stimulated the growth of livestreaming e-commerce. In 2020, China’s livestreaming e-commerce market exceeded ¥1.2 trillion, with an annual growth of 197.0% (IResearch, 2021). Livestreaming shopping has become a new engine of economic growth in China (Ma, 2021). According to CNNIC (2022), there were 1.032 billion Chinese netizens at the end of 2021, and 99.7% of these netizens were mobile device users. By the end of 2020, there were 388 million e-commerce livestreaming users, accounting for 39.2% of the total netizens (China Live E-Commerce Industry Research Report, 2021). A large number of netizens provides a vigorous driving force for the development of e-commerce. The sales volume during the “618” promotion period (1–18 June) of Jingdong livestreaming increased by 161% year-on-year (CNNIC, 2022). The first-hour sales volume on Taobao livestreaming on 1 June 2021 exceeded the whole-day sales volume for the same day in 2020 (Sina, 2021). The Gross Merchandise Value (GMV) of major B2C e-commerce platforms in China during the Double 11 period in 2021 was ¥952.3 billion, to which livestreaming shopping contributed over ¥73 billion (Syntun, 2021).

According to eMarketer’s Global E-Commerce Report, China continued to lead the global e-commerce market in 2021 with 792.5 million digital buyers (33.3% of the global total) and $2.779 trillion e-commerce sales (56.8% of the global total). The e-commerce share of total retail sales in China is the highest compared to other countries. China has become the first country to account for more than 50% of total transactions through e-commerce retail sales (Ethan Cramer-Flood, Global Ecommerce Update, 2021). Live commerce or live video shopping generated sales of $171 million in 2020 in China (Utsi, 2022). Compared to China, the United States and Europe are taking baby steps in the expansion of livestreaming commerce. Amazon and YouTube advanced capabilities of their websites and reviewing consumers’ reaction toward livestream shopping (Ryan, 2020). Livestreaming e-commerce generated $60 billion sales in 2019 globally, but the US market contributed less than $1 billion (Kharif and Townsend, 2020). However, the US market is growing fast, especially in certain products, for instance, apparel, makeup, and alcoholic beverages (Kharif and Townsend, 2020). In the European market, few consumers understand the concept of live video shopping, which is one of the main reasons why live commerce is not as popular as in the Chinese market (Andersson and Pitz, 2021). Live video service providers Zellma and Bambuser^1^ suggest that companies in Europe need education on how to apply livestreaming e-retailing into their business, and they are confident that European consumers are ready to embrace new online shopping forms (Andersson and Pitz, 2021). In 2021, an online survey conducted in Poland, Spain, France, and the United Kingdom reported that 7,261 respondents were interested in livestreaming on e-commerce website/app and 6,602 were interested in social media livestreaming (Forrester, 2021). Hence, investigation would provide insight into how e-tailers promote featured products on live commerce platforms in China, and how consumers perceive this marketing.

E-commerce livestreaming shopping re-establishes the relationship between merchants, commodities, and consumers (Liu, 2021). In a livestreaming shopping room, the anchor creates an immersive experience for consumers (Luo et al., 2020) and stimulates impulsive purchase through a series of strategies (Xu et al., 2020). In e-commerce livestreaming shopping, it takes only a moment for consumers to be attracted by live product promotion introduced by anchors regardless of whether consumers are hedonistic or utilitarian in outlook (Xu et al., 2020). Triggered consumption behavior is mostly impulsive purchase (Li, 2020). According to the iMedia Research report “User Research and Analysis of China’s Live Streaming E-commerce in the First Half of 2020,” 65.2% of livestreaming viewers purchased goods in the livestreaming shopping room, and 49.5% admitted that their purchases were impulsive (IMedia Research, 2020). A recent study on online purchase intention in China asserts that online shopping in the social commerce setting is driven more by hedonistic than utilitarian motivation (Akram et al., 2021), and that impulsive buying contains hedonic features (Akram et al., 2018b). IMedia Research (2020) considered that the most direct support for the incremental performance of e-commerce is to trigger more consumers to purchase impulsively for unplanned needs when watching livestreaming. Awakening unplanned consumption is a long-term and deep driving force for e-tailers using livestreaming. Thus, this study investigated the practical significance of impulse purchase behavior in e-commerce live broadcasts to inform marketing strategy aimed at impulsive buying.

Good sales performance is inseparable from effective marketing strategy (Akram et al., 2018a; Varadarajan, 2020). The continuing growth of livestreaming shopping makes it important for e-commerce investors and managers to understand influencing factors for impulse buying in livestreaming shopping. Livestreaming e-commerce aims to sell products and services to consumers (Hu and Chaudhry, 2020; Wongkitrungrueng et al., 2020). This business model contains the basic elements of the “People-Product-Place” theory essential in retailing (Guo and Xu, 2021), but in different forms. E-commerce livestreaming shopping reconstructs a retail scenario comprising “People-Product-Place” to realize the real-time, situationalization and visualization of communication in the entire process of e-commerce livestreaming shopping, and to bring remarkable features of strong interactivity and authenticity (Duan, 2020). Researchers have suggested that anchor promotion, product promotion, and livestreaming atmosphere are likely to trigger strong emotions in consumers, leading to impulsive purchases (Xu et al., 2019, 2020; Lee and Chen, 2021; Ming et al., 2021). Empirical studies find that online consumers are easily triggered by marketing stimuli to make impulsive purchases, and rich marketing methods help consumers avoid monotony and frustration, thus enhancing the shopping experience (Sundström et al., 2019).

Purchase behavior in live-broadcast shopping rooms has become a popular research topic. Researchers have investigated external stimuli such as atmosphere clues (Gong et al., 2019), IT affordances (Dong and Wang, 2018), discounted prices and scarcity (Wang S. Q., 2021; Yan, 2021); and inherent characteristics of livestreaming marketing, including the attributes of anchors (Han and Xu, 2020), their communication styles (Wu N. et al., 2020), their identity and information source characteristics (Liu F. J. et al., 2020), relationship ties and customer commitment (Peng et al., 2021); opinion leaders (Yin, 2020; Lakhan, 2021); interaction (Wang S. Q., 2021; Yan, 2021); perceived enjoyment (Lakhan, 2021; Lee and Chen, 2021); and perceived product usefulness (Lee and Chen, 2021). There are few studies on the impact of livestreaming marketing strategies on impulsive purchase. Livestreaming is jointly constructed by various stakeholders, including those from the three retail marketing elements “People-Product-Place.” Clearly, effective multiparty relationships are central to any effective livestreaming marketing strategy.

In this context, based on stimulus-organism-response (SOR) theory, this study introduced the “People-Product-Place” marketing strategies for livestreaming from the perspective of consumer perception, to study how livestreaming influences impulsive purchasing. This study aimed to more comprehensively explain the perspective for research on the impact of consumer impulsive purchase behavior. As well as to enhance the understanding of the “People-Product-Place” marketing model of e-commerce livestreaming, guide marketers to improve the profitability of livestreaming e-commerce, and provide reference for the healthy and sustainable development of e-commerce livestreaming industry. Then, hopefully to provide reference of economic recovery under the impact of the normalization of the COVID-19 epidemic for many other countries.

The rest of the article is structured as follows: the theoretical framework and hypothesis development are discussed in the following section. Research design and methodology, and data analysis and hypotheses testing are described in the subsequent sections. The findings and their implications, study limitations, and further research are discussed in the final section.

## Theoretical Framework and Hypothesis Development

### Theoretical Framework

SOR theory (Mehrabian and Russell, 1974) underpins the study. *Stimulus* refers to external stimuli; *Organism* represents the internal state of an individual when that individual perceives a stimulus; and *Response* is the behavior of the individual in response to stimuli. The SOR model is a mediation model in which a stimulus provokes a response through the mediating effects of the organism. The SOR model has been applied to studies of online purchasing behavior (Hashmi et al., 2019; Huang and Suo, 2021; Karim et al., 2021; Lee and Chen, 2021; Ming et al., 2021). Impulse purchases are unplanned and occur when consumers are stimulated internally and/or externally (Rook, 1987; Lim et al., 2017). Piron (1991) believed that stimuli can come from products, the shopping environment, or the people who accompany you for shopping. This is consistent with the elements “People-Product-Place.” Stimuli in livestreaming have some similar and some different characteristics with traditional online and offline shopping (Gong et al., 2019). This study used “People-Product-Place” as the stimulus factor (S), involvement as internal state of an individual (O), and explored the effect on impulsive purchase behavior (R).

### Hypothesis Development

#### Impulsive Purchase Behavior

Impulse buying is a popular subject in the domain of consumer decision-making. Researchers claim that impulse buying accounts for 40–80% of all purchases (Rodrigues et al., 2021). Lee and Chen (2021) stress that instant reactivity and convenience trigger impulsive purchase in mobile commerce. Studies have identified products that are bought impulsively, including groceries (Inman et al., 2009; Bellini et al., 2017), financial products (Lučić et al., 2021), milk tea (Guo et al., 2017; Wu et al., 2021), necessities during COVID-19 pandemic (Islam et al., 2021), “unhealthy” foods (Verplanken et al., 2005), and brand-related user-generated content products (Kim and Johnson, 2016). Some studies are keen to discover how different purchase channels influence impulsive buying, for instance, online markets (Kim and Johnson, 2016; Guo et al., 2017; Aragoncillo and Orus, 2018; Pal, 2021; Rejikumar and Asokan-Ajitha, 2021; Wu et al., 2021), mobile commerce (San-Martin and López-Catalán, 2013; Chen et al., 2021), and offline/in-store shopping (Rook and Fisher, 1995; Inman et al., 2009; Tendai and Crispen, 2009; Sharma et al., 2010; Aragoncillo and Orus, 2018). However, few studies focus on impulsive purchase through the livestream shopping channel (Lee and Chen, 2021; Wang S. Q., 2021).

There are various definitions of impulse buying. Rook (1987) describes impulse buying as spontaneous and hedonic purchase driven by an urgent, forceful, and persistent craving, regardless of possible consequences. Lučić et al. (2021) assert that impulsive purchase is actuated by irrational emotions. Researchers also claim that impulsive purchase is the result of an irresistible reaction that is triggered by often deliberately designed stimuli (Stern, 1962; Rook, 1987; Liu et al., 2013; Kim and Johnson, 2016; Aragoncillo and Orus, 2018). Aragoncillo and Orus (2018) summarized and categorized features that induce impulsive purchase in offline and online channels, from previous studies. They argue that “greater product assortment and variety, sophisticated marketing techniques, credit cards, anonymity, lack of human contact, and easy access and convenience are the encouraging factors to online impulsive purchase” (Aragoncillo and Orus, 2018, p. 47). Akram et al. (2017) believe that “impulsive purchasing is an immediate, unplanned, compelling, and sudden purchase behavior while shopping” (p. 76) (Akram et al., 2017). When comparing characteristics of online store to livestreaming shopping, it is not difficult to notice that livestreaming shopping contains all the encouraging factors mentioned and offers higher levels of stimuli. Specifically, payment is made easier by biometric fingerprint scanning on smart mobile devices, bypassing typed-in credit card passwords. Livestreaming provides for more comprehensive product display by e-commerce anchors than predesigned images and text on webpages. In livestreaming shopping rooms, there is instant interaction and sharing of product experience between anchors and customers, and between customers. Free shipping and unconditional returns or refunds encourage impulse buying. Big data analytics facilitate tailor-designed promotions and accurate/precise targeting of individual consumers. Therefore, based on discussion above, this study assumes that marketers use effective strategies to stimulate impulse buying on livestreaming shopping platforms. Specific constructs of interest include perceived e-commerce anchor attributes, perceived scarcity, immersion, and involvement.

#### Marketing Strategies Applied to Livestreaming E-commerce

**FIGURE 1:**
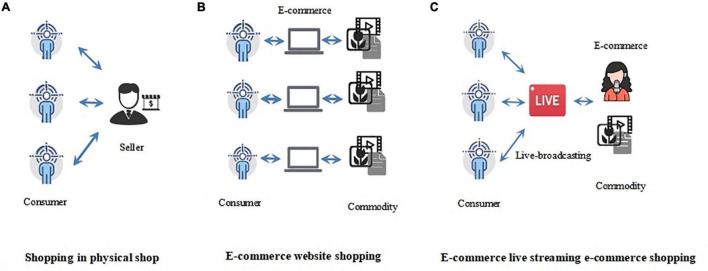
Three types of shopping experience.

The success of livestreaming retailing lies in its high interactivity, entertainment value, authenticity, and visibility (Bründl et al., 2017). As shown in Figure 1, livestreaming (Figure 1C) is different from shopping in a physical store (Figure 1A) and online shopping (Figure 1B) because consumers can watch, interact, comment, and purchase using a mobile device anywhere and anytime (Liu L. et al., 2020). Traditional online shopping is search-based, requiring searching, comparing, and choosing before purchasing. Traditional online retailing thus relies on consumer initiative, and retailing success relies to a significant extent on consumers looking for products, with clear objectives in mind (Virdi et al., 2020). In livestreaming retailing, consumers are guided by anchors who actively promote products to them (Bründl et al., 2017; Ang et al., 2018). Figure 1 illustrates the three types of shopping experience:

Marketing strategy is the most direct embodiment of merchants facing consumers. Effective marketing strategy in retailing is to provide effective stimuli for buying (Zhu et al., 2019), and specific stimuli often lead to impulse buying (Floh and Madlberger, 2013). In traditional e-commerce, users browse the goods on a shopping platform and typically spend considerable amounts of time considering purchase: this is “decision-making consumption” (Rezaei, 2015). In livestreaming, consumers are provided with various designed entertainments. Whether they are hedonic or utilitarian consumers are readily attracted by personal charm, product introduction, promotion information, and livestreaming scenes deliberately designed to lead to consumption behavior (Liu F. J. et al., 2020; Xu et al., 2020). Impulsive purchase is awakened by marketing strategies planned by anchors.

The success of livestreaming retailing lies in the good coordination of the elements “People-Product-Place” (Luo et al., 2020), which is a perspective that should not be ignored when studying marketing strategy (Wang K., 2021). “People” represents the anchor, who is the key factor to attract “followers” to watch. Anchor attributes is an important influencing factor in purchase decisions in livestreaming shopping Liu F. J. et al., 2020; Meng et al., 2020; Xu et al., 2020). Anchor attributes marketing becomes one of the major streams in livestreaming e-commerce by introducing products to their audience (Liu, 2021). “Product” represents promoted goods recommended by the anchor. The main marketing method for “goods” is hunger marketing through the creation of availability stimuli, “limited time,” and “limited quantity.” The anchor creates the phenomenon of “short supply” that increases consumer perceived time pressure and product scarcity, stimulating impulsive purchase (Gupta and Gentry, 2019; Cheng et al., 2020). “Place” is the final presentation of the e-commerce livestreaming scene. With the support of “people” and “product,” the internet platform built a communication scene integrating shopping, livestreaming, communication, and other functions. In the broadcast room, the extraordinary sense of temporal presence allows participants to sink into the immersive experience. Immersive marketing develops consumption into an “entertainment game” of shopping (Luo et al., 2020). Therefore, we studied the influence of three elements in marketing strategy on impulsive purchase: anchor attributions, hunger marketing, and immersive marketing.

##### Perceived E-commerce Anchor Attributes and Impulsive Purchase Behavior

E-commerce anchors are the core of marketing strategy in livestreaming retailing. An e-commerce anchor is one who introduces and displays products comprehensively to customers (Zhu et al., 2021). Unlike traditional television broadcasters, e-commerce anchors provide guidance to customers by sharing experiences based on their own consumption of the promoted products, answer viewers’ questions in real time, and interact with viewers based on their requests, and display products in ways that static images and texts cannot (Sun et al., 2019; Han and Xu, 2020). Research has shown that the attributes, features, or characteristics of e-commerce anchors significantly influence purchase decisions or impulsive purchase on livestream shopping platform (Li, 2021; Zhao and Feng, 2021; Zhu et al., 2021). Zhu et al. (2021) classify anchors’ characteristics into physical attractiveness, professional ability, and social attractiveness. Zhao and Feng (2021) assert that interactivity, professionalism, and charisma are important characteristics of e-commerce anchors who are opinion leaders. Their findings indicate that anchor characteristics positively influence consumer purchase intention. In their qualitative study, Han and Xu (2020) interviewed 68 livestreaming shoppers and summarized the attributes of e-commerce anchors. These authors argue that charisma, recommendation attributes, and display and interaction attributes are essential attributes of an e-commerce anchor. In summary, the literature discusses important attributes of e-commerce anchors, but studies on how these attributes influence impulsive purchase behavior are still limited. In addition, because of recent tax evasion by several famous livestream anchors in China, the essential requirements for becoming e-commerce anchors have been raised. As discussed, consumers’ perception of e-commerce anchor attributes is important. In this study, these attributes are defined in terms of how consumers/viewers perceive the presented image of an anchor. These attributes consist of whether the anchor observes discipline and law, his/her communication and professional skills, and whether consumers find the anchor reliable and have professional knowledge on the products being promoted. Thus, this study proposes the following hypothesis:

H1a: Perceived e-commerce anchor attributes have a positive effect on impulsive purchase behavior.

##### Perceived Scarcity and Impulsive Purchase Behavior

Studies show that perceived scarcity significantly affects panic buying (Islam et al., 2021; Li et al., 2021) and influences decision-making in impulse buying (Wu et al., 2021). By deliberately manipulating the supply of products, anchors create an ambiance of the shortage of goods in livestreaming shopping. In this study, perceived scarcity is intentional creation of limited time and quantity scarcity by anchors in livestreaming shopping (Aggarwal et al., 2011; Gupta and Gentry, 2019; Islam et al., 2021). Following introduction of product functions, quality, and any other information consumers need to know, anchors specifically emphasize the limited availability of the products for on-the-spot purchase, especially when viewers significantly outnumber product units. Anchors also magnify the countdown process, which signals urgency to buy as soon as the countdown ends. Such a situation creates perceived product scarcity and competitive purchase pressure (Guo et al., 2017).

Perceived scarcity has been studied as an independent variable (Guo et al., 2017; Akram et al., 2018b; Gupta and Gentry, 2019; Islam et al., 2021; Wu et al., 2021), a mediating factor (Li et al., 2021), and a moderating factor (Cheng et al., 2020) purchase decision, panic buying, urgency to buy, or impulse buying. These studies indicate that perceived scarcity positively influences panic buying (Li et al., 2021) and indirectly influences panic or impulse buying through perceived arousal (Guo et al., 2017; Islam et al., 2021; Wu et al., 2021). Perceived scarcity is shown to strongly predict online impulsive buying in Chinese social commerce (Akram et al., 2018b). Interestingly, studies find in in-store consumers, perceived scarcity does not directly affect urgency to buy, but perceived scarcity triggers in-store hording behavior (e.g., holding the clothes in hand while shopping) and in-store hiding behavior (e.g., hiding clothes somewhere else other than the place they should be) (Gupta and Gentry, 2019). These authors assert that the relationship between perceived scarcity and urgency to buy is mediated by anticipated regret (Gupta and Gentry, 2019). A moderating effect of perceived scarcity was not found in Cheng et al.’s (2020) study. Because the literature reports various results and inconsistent findings, this study aimed to contribute more evidence on perceived scarcity as both a direct and indirect influencing factor on impulsive purchase. Thus, this study postulates the following hypothesis:

H1b: Perceived scarcity has a positive effect on impulsive purchase behavior.

##### Immersion and Impulsive Purchase Behavior

In a study about viewers’ complete absorption of co-viewing experience on video websites, immersion is described as a joyful feeling that one is deeply absorbed in a mediated world, meanwhile forgetting or failing to pay attention to people or environment around him/her (Fang et al., 2018). In a virtual reality environment, the use of augmented reality is expected to give users a higher level of immersion. In this context, immersion is described as a complete engrossing feeling of neglecting the actual environment (Yim et al., 2017). Hudson et al. (2019) argue that individuals’ perceived levels of immersion differ, hence, their study focused on subjectively experienced immersion. Previous research has focused mainly on the mediating role of immersion in various activities. For instance, Yang et al. (2021) examined the mediating effect of immersion between social presence and customer loyalty intentions toward recommendation vlogs. Their findings confirm the proposed hypothesis and indicate that increased consumer immersion positively influences customer loyalty. In a study of try-on experience of wrist watches with augmented reality, Song et al. (2020) found that immersion mediates the relationship between environmental embedding and feelings of ownership. Immersion and perceived benefit have been found to mediate between social presence and customer loyalty on co-viewing experience in video websites (Fang et al., 2018). In a study of fashion product purchase intention, immersion was studied as a mediator between five characteristics of fashion influencers and their followers’ purchase intentions (Kim et al., 2021). Evidence that immersion in augmented reality positively affects online tourists’ willingness to pay was found in a study about AR tourism destination experience, without highlighting the mediating role of immersion (Huang, 2021).

The literature indicates that the immediate relationship between immersion and impulsive purchase is rarely studied. A study of interactive marketing (Kabadayi and Gupta, 2005) showed that deeply immersed customers tend to indulge in longer hours of digital media. A study in Taiwan provides evidence that high level of the absorbed-in state greatly influences unplanned buying online, and consumers are willing to pay more in such situations (Niu and Chang, 2014). Thus, this study defines immersion as a joyful state of being absorbed and engrossed, losing awareness of time and forgetting about one’s surroundings when watching livestream promotions. The following hypothesis was proposed:

H1c: Immersion has a positive effect on impulsive purchase behavior.

#### Involvement

Involvement is an important variable affecting consumers’ purchase decision-making. Zaichkowsky (1985, p. 32) defines involvement as “a person’s perceived relevance of the object based on inherent needs, values, and interests” and suggests that this definition could be applied to research on purchase decisions. Much research has evaluated “product” involvement in purchase decisions. For instance, Cheng et al. (2020) adapted Zaichkowsky’s (1985) 10 measurement dimensions to evaluate product involvement in livestreaming shopping. A study on online ordering behavior for food delivery measured product involvement based on nine external cues for action. These external cues are mainly concerned with nine different aspects of safety and customer rating, including food safety, advertisement safety, and safety of food retailers (Mehrolia et al., 2020). A study on purchase decisions of halal products measured product involvement in two aspects: consumer perceptions of the degree of importance of targeted products and the number of attributes of a halal product that consumers regard as imperative (Rachmawati et al., 2020).

To measure the involvement construct more comprehensively, some researchers extend the definition of involvement by including more aspects of involvement besides product attributes. In a study of customer satisfaction in mobile commerce, “buying-selling environment” was included as a measurement item for involvement (San-Martin and López-Catalán, 2013). Mou et al. (2019) studied the influence of product involvement, characterized as cognitive and affective involvement, and platform involvement, characterized as enduring and situational involvement, on consumer purchase intention in cross-border e-commerce. Based on the foregoing discussion, this study defines *involvement* as a variable that includes aspects of consumer’s interests and valuation of promoted products and services, and perceived relevance and importance of the shopping environment (livestreaming shopping).

#### Marketing Strategies and Involvement

To create strong bonding between consumers and shopping platform, e-commerce managers have worked on measures to make consumers feel connected or intrigued. E-commerce managers design marketing to attract, retain, and connect viewers in livestreaming shopping, with the aim of increasing their involvement, and inducing impulse buying. In the literature, antecedents of involvement include product description (Mou et al., 2019) and innovativeness in new technology (San-Martín et al., 2017). In the domain of livestreaming shopping, there are likely to be more antecedents of involvement to be discovered. Thus, this study postulates the following hypotheses:

H2a: Perceived e-commerce anchor attributes positively affect involvement.

H2b: Immersion positively affects involvement.

H2c: Perceived product scarcity positively affects involvement.

#### Involvement and Impulsive Purchase Behavior

Studies have also examined the direct impact of involvement on behaviors such as consumer satisfaction, purchase intention, or decision-making (San-Martin and López-Catalán, 2013; Mehrolia et al., 2020; Rachmawati et al., 2020). Several studies provide evidence that high involvement induces purchase intentions/decisions (San-Martin and López-Catalán, 2013; Siala, 2013; Mehrolia et al., 2020; Rachmawati et al., 2020). Thus, this study postulates the following hypothesis:

H3: Involvement positively affects impulsive purchase behavior.

#### The Mediating Role of Involvement

Involvement has been studied as a mediating factor between product description and purchase intention in cross-border e-commerce. Three out of four dimensions of involvement are found to mediate between product description and purchase intention in that study (Mou et al., 2019). To contribute empirical evidence for the mediating effect of involvement between relationships various variables and impulsive purchase, this study postulates the following hypotheses:

H4: Involvement mediates the relationship between marketing strategies applied in livestreaming shopping room and impulsive purchase behavior.

H4a: Involvement mediates the relationship between perceived e-commerce anchor attributes and impulsive purchase behavior.

H4b: Involvement mediates the relationship between immersion and impulsive purchase behavior.

H4c: Involvement mediates the relationship between perceived scarcity and impulsive purchase behavior.

## Research Design and Methodology

This study adopts a positivist paradigm. Data were collected online using self-reporting questionnaires, and structured equations were used to evaluate the relationship between variables and to test study hypotheses. This topic focuses on the research on the influence mechanism of consumers’ impulsive purchase behavior in the context of livestreaming e-commerce. To ensure the validity of the research results, people who have had an online livestreaming shopping experience are selected as the target group, which is conducive to objectively assessing consumers’ impulsive purchase behavior on livestreaming e-commerce.

### Research Design

**FIGURE 2:**
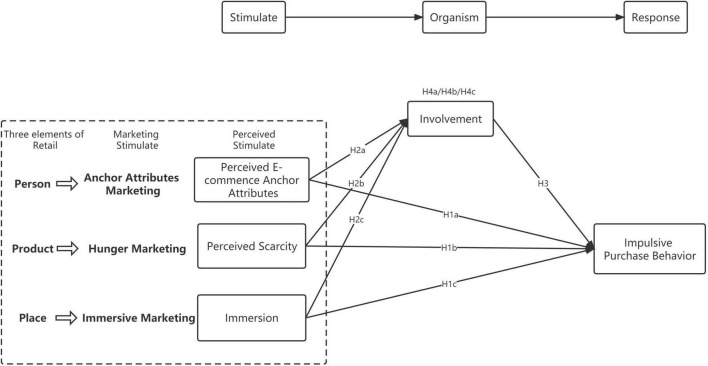
Research model.

The study focuses on the marketing-generated stimuli that influence impulse purchase in livestreaming retailing: anchor attributes, hunger marketing, and immersive marketing. *Perceived anchor attributes*, *perceived scarcity*, and *immersion* are independent variables; *impulsive purchase behavior* is the dependent variable; and *involvement* is a mediator. Figure 2 shows the framework that links these variables.

### Instrument Development

The study constructed a SOR model and attempted to explain consumer impulsive purchase from a psychological and behavioral perspective. Data were collected through an online self-administered questionnaire. To ensure validity reliability of measurement of the variables, the study defined these variables, and identified and adapted/modified measurement items from the literature to fit the context and object of the study from the literature. All the measurement items were rated on a 5-point Likert scale anchored on 1 (strongly disagree) and 5 (strongly agree). Table 1 shows the measurement items.

**TABLE 1:** Measurement items.

Construct	Measurement items	Researchers
E-commerce anchor attribute	The e-commerce anchor observes disciplines and obeys laws.	Wang S. Q., 2021; Zhu et al., 2021
	The e-commerce anchor has professional skills.	
	The e-commerce anchor is reliable.	
	The e-commerce anchor has strong communication skills.	
	The e-commerce anchor has good expertise on the promotional products.	
Immersion	I always feel time flies when watching product promotion in live streaming shopping room.	Yang et al., 2021
	Watching anchors promote goods in live streaming shopping room makes me forget my surroundings.	
	I was absorbed as watching anchors promote goods in live streaming shopping room.	
	I felt joyful watching anchors promote goods in live streaming shopping room.	
Perceived Scarcity	As watching live streaming shopping promotion, I think many people would compete with me to buy the promotional items.	Cheng et al., 2020; Li et al., 2021
	As watching live streaming shopping promotion, I think the promotional product would sell out fast.	
	I think that product scarcity is strategically created by anchors in live streaming shopping scene.	
Involvement	I am very interested in the products and services offered over the live streaming shopping.	San-Martin and López-Catalán, 2013; Mou et al., 2019; Cheng et al., 2020
	The products promoted in live streaming shopping room are important to me.	
	My level of involvement with the products and services offered over the live streaming shopping is high.	
	I am particularly involved with live streaming shopping buying-selling environment.	
Impulsive purchase behavior	As I watch live streaming shopping, I often purchase items other than or in addition to my specific shopping goal.	Aragoncillo and Orus, 2018; Chen et al., 2021; Lučić et al., 2021
	There are a lot of things that I have bought from live streaming shopping, but I rarely use them.	
	I barely purchase unplanned products from live streaming shopping. (reverse)	
	I sometimes buy things from live streaming shopping because I like buying things, rather than because I need them.	

The online questionnaire comprised three parts: an introduction describing the purpose of the questionnaire, to dispel respondents’ concerns, and to enable respondents to recall live e-commerce shopping by describing live shopping. The second part comprised the questionnaire that measured perceived anchor attributes, perceived scarcity, immersion, and impulsive purchase behavior. The third part elicited respondent demographics, i.e., gender, age, education level, income, and occupation.

### Data Collection

We conducted online questionnaire survey by using Questionnaire Star^2^, which is a platform with 2.6 million database members, covering more than 90% of universities and research institutions in China. Convenience sampling was used, and we elicited voluntary responses from individuals with experience of shopping in live e-commerce. We asked respondents to respond to the items based on their live e-commerce shopping experience. Screening of the 456 response sets received yielded 437 sets for analysis, giving an effective response rate of 94.96%.

Table 2 shows the demographic characteristics of respondents.

**TABLE 2:** Demographic data of respondents.

	Category	Frequency	Percentage (%)
Gender	Male	89	20.4%
	Female	348	79.6%
Age	18–30	228	52.2%
	31–40	99	22.7%
	41–50	99	22.9%
	>50	11	2.5%
Education level	Primary and secondary school	11	2.5%
	High school and equivalent	24	5.5%
	Undergraduate and equivalent	329	75.3%
	Post graduate	73	16.7%
Monthly income (RMB, Yuan)	≦2,000	181	41.4%
	2,001–4,000	64	14.6%
	4,001–6,000	75	17.2%
	≥6,001	117	26.8%
Occupation	Student	183	41.9%
	Civil servant, employee of state organization or public institution	103	23.6%
	Employees of corporation	80	18.3%
	Others	71	16.2%

Women accounted for 79.6% of respondents, a relatively large percentage. In four age groups, participants are mainly between 18 and 30 years, reaching 52.2 and 97.5% of participants are younger than 50 according to the statistics. This shows that most of the people participating in live shopping are younger groups, and they are more likely to embrace new shopping channels. From the perspective of education level, 80.8% have a bachelor’s degree or above, indicating that the participants’ education level is relatively high. The highest monthly income is less than or equal to 2,000, accounting for 41.4%, and the proportion of students is 41.9%, which is consistent with the proportion of monthly income less than or equal to 2,000. This suggests that the young student group is the backbone of livestreaming shopping.

### Data Analysis

SPSS and AMOS statistical software were used to analyze the data. Confirmatory factor analysis (CFA) was used to test the reliability validity of the measurement model. We then verified the model using structural equation modeling (SEM) in AMOS software, using regression analysis to analyze the relationship between variables, and bootstrapping to test the hypothesis of the mediating effect.

## Results and Findings

### Construct Validity and Reliability

CFA is usually used to test data reliability and validity of data to evaluate questionnaire quality (Hou et al., 2004). In this study, AMOS software was used to establish a measurement model with five latent variables, including three independent variables, one dependent variable, and one mediator, and CFA was conducted. The test results of the measurement model are shown in Tables 3–5.

**TABLE 3:** Goodness-of-fit statistics of the measurement model.

Goodness of fit statistic	Criterion	Source	Value	Information
Chi-square fit statistics/degree of freedom (CMIN/DF)	≤5	Marsh and Hocevar, 1985	1.79	Good
Comparative fit index (CFI)	≥0.9	Byrne, 2013	0.971	Good
Goodness of fit index (GFI)	≥0.9	Baumgartner and Homburg, 1996	0.939	Good
Tucker-lewis index (TLI)	≥0.9	Bentler, 1983	0.966	Good
Normed fit index (NFI)	≥0.9	Bentler and Bonett, 1980	0.911	Good
Root mean square error of approximation (RMSEA)	≤0.1	MacCallum et al., 1996	0.043	Good

**TABLE 4:** Reliability and validity results of measurement mode.

	Factor loadings	Cronbach’s alpha	CR	AVE
Perceived Anchor Attributes	0.708	0.852	0.8561	0.5464
	0.752			
	0.594			
	0.813			
	0.807			
Perceived Scarcity	0.807	0.85	0.8527	0.6591
	0.859			
	0.622			
Immersion	0.622	0.832	0.8376	0.5675
	0.883			
	0.689			
	0.767			
Involvement	0.701	0.837	0.8389	0.5666
	0.811			
	0.783			
	0.71			
Impulsive purchase behavior	0.764	0.87	0.8724	0.6321
	0.86			
	0.833			
	0.715			

**TABLE 5:** Analysis of discriminant validity.

Variable	Perceived anchor attributes	Perceived scarcity	Immersion	Involvement	Impulsive purchase behavior
Perceived anchor attributes	0.739				
Perceived scarcity	0.532***	0.753			
Immersion	0.297***	0.171***	0.812		
Involvement	0.513***	0.545***	0.424***	0.753	
Impulsive purchase behavior	0.431***	0.689***	0.342***	0.635***	0.6321

***p < 0.01. The square root of average variance extracted (AVE) is shown on the diagonal of the matrix.

The indices of fit (Table 3) for the measurement model were chi-square fit statistics/degree of freedom (CMIN/DF) = 1.79, comparative fit index (CFI) = 0.971, goodness of fit index (GFI) = 0.939, TFI = 0.966, normed fit index (NFI) = 0.9011, and root mean square error of approximation (RMSEA) = 0.043. According to the criteria in Table 3, the model fit is good. As shown in Table 4, individual item loadings are required to be above 0.50 for adequate and Cronbach’s alpha and CR value are higher than the recommended value of 0.700. The average variance extracted (AVE) scores were higher than the recommended value of 0.500, indicating the internal consistency and component reliability of each variable are good. Table 3 also shows that inter-construct correlations are all smaller than the square root of AVE, indicating the data have good discriminant validity. The reliability and validity of the data were therefore good and suitable for further analysis.

### Hypothesis Testing

Before hypothesis testing, degree-of-fit testing is carried out to test the relationship between variables in the structural model. The model fit statistics were CMIN/DF = 2.573, CFI = 0.942, GFI = 0.914, TFI = 0.932, NFI = 0.942, and RMSEA = 0.06. According to the judgment criteria shown in Table 3, the model fit is good.

#### Path Coefficient Testing

AMOS software was used to test path coefficients to verify the hypothesis of direct relationship. Table 6 shows the standardized path coefficients.

**TABLE 6:** Results of direct effects.

H#	Path			Coefficient	C.R.	*P*	Result
H1a	Perceived anchor attributes	→	Impulsive purchase behavior	0.122	2.583	0.01***	Supported
H1b	Perceived scarcity	→	Impulsive purchase behavior	0.105	2.318	0.02***	Supported
H1c	Immersion	→	Impulsive purchase behavior	0.522	8.761	***	Supported
H2a	Perceived anchor attributes	→	Involvement	0.322	6.013	***	Supported
H2b	Perceived scarcity	→	Involvement	0.236	4.658	***	Supported
H2c	Immersion	→	Involvement	0.469	8.204	***	Supported
H3	Involvement	→	Impulsive purchase behavior	0.273	4.377	***	Supported

***p < 0.01.

In the relationship between consumer-perceived marketing strategy and impulsive purchase behavior, *perceived anchor attributes* (β = 0.122, *p* = 0.01), *immersion* (β = 0.522, *p* = 0.000), and *perceived scarcity* (β = 0.105, *p* = 0.02) positively influence impulsive purchase behavior, supporting H1a, H1b, and H1c. In the relationship between consumer-perceived marketing strategy and impulsive purchase behavior, *perceived anchor attributes* (β = 0.322, *p* = 0.000), *immersion* (β = 0.469, *p* = 0.000), and *perceived scarcity* (β = 0.236, *p* = 0.000) positively influence impulsive purchase behavior, supporting H2a, H2b, and H2c. In the relationship between involvement and impulsive purchase behavior, *involvement* (β = 0.273, *p* = 0.000) positively influenced impulsive purchase behavior, supporting H3.

#### Testing for Mediating Effect

In the bootstrap method to detect a mediating effect, the bootstrap iteration was set to 2,000 times. Whether the mediating effect is significant is judged by whether the 95% confidence interval contains 0. The three mediation paths are *perceived anchor attributes → involvement → impulsive purchase behavior*, *immersion → involvement → impulsive purchase behavior*, and *perceived scarcity → involvement → impulsive purchase behavior;* the analysis results are shown in Table 7, as follows:

**TABLE 7:** Results of mediating effects.

H#	Path	Total effects			Indirect effects			Direct effects			Result
		Bias-corrected percentile 95%CI			Bias-corrected percentile 95%CI			Bias-corrected percentile 95%CI			
		Lower	Upper	*P*-value	Lower	Upper	*P*-value	Lower	Upper	*P*-value	
H4a	Perceived anchor attributes→involvement→impulsive purchase behavior	0.091	0.329	0.001 ***	0.041	0.16	0.000 ***	0.005	0.245	0.039 **	Supported. partial mediation
H4b	Perceived scarcity→involvement→impulsive purchase behavior	0.055	0.279	0.002 ***	0.025	0.117	0.000 ***	-0.015	0.213	0.08	Supported. full mediation
H4c	Immersion→involvement→impulsive purchase behavior	0.551	0.732	0.001 ***	0.063	0.204	0.001 ***	0.404	0.636	0.001 ***	Supported, partial mediation

***p < 0.01, **p < 0.05.

In the path *perceived anchor attributes → involvement → impulsive purchase behavior*, the 95% confidence intervals [0.041, 0.16] did not include 0, *p* = 0.000. The mediating effect was thus significant, supporting H3a. The 95% confidence intervals for total and direct effects were respectively, [0.091, 0.329] and [0.005, 0.245]; both sets of intervals did not contain 0, so *involvement* partially mediates the relationship between *perceived anchor attributes* and impulsive purchase behavior.

In the path *perceived scarcity → involvement → impulsive purchase behavior*, the 95% confidence intervals [0.025, 0.117] do not contain 0, *p* = 0.000, indicating that the mediation effect is significant and H3b is supported. The 95% confidence intervals of total effect [0.055, 0.279] do not contain 0, but the 95% confidence interval of direct effect [-0.015, 0.213] contains 0, so *involvement* fully mediates the relationship between *perceived scarcity* and impulsive purchase behavior.

In the path *immersion → involvement → impulsive purchase behavior*, the 95% confidence intervals of total effect [0.063, 0.204] do not contain 0, *p* = 0.001, indicating it is significant. The 95% confidence intervals of total and direct effects [0.551, 0.732] and [0.404, 0.636], respectively, both do not contain 0. Therefore, *involvement* partially mediates the relationship between *immersion* and impulsive purchase behavior.

## Discussion and Implications

### Discussion

Based on SOR theory, this study used a framework of “marketing stimulus—involvement—impulsive purchase behavior” to investigate consumer impulsive purchase behavior in livestreaming shopping. By introducing the “People-Product-Place,” three elements of marketing stimulus as a marketing strategy of the livestreaming shopping platform, this study investigates the influencing mechanism and examines the influencing effects of perceived e-commerce anchor attributes, perceived scarcity, and immersion on consumers’ impulsive purchase behavior based on user perception perspective. Moreover, this study also examines the mediating effect of involvement between the three stimulus elements and consumers’ impulsive purchase behavior. Therefore, to explain the influencing mechanism of different marketing strategies in the livestreaming room on consumers’ impulsive purchase behavior. Findings of this research are discussed as follows.

Perceived e-commerce anchor attributes, perceived scarcity, and immersion positively influence impulsive purchase behavior. These findings are similar to previous studies, which report that e-commerce anchor attributes (Li, 2021) and scarcity influence impulsive purchase behavior (Akram et al., 2018b). This study indicates that building e-commerce anchor attributes, creating pressure to purchase, and developing immersion are important and effective measures to trigger impulse buying. This finding is similar to previous studies that claim that sales promotion significantly affects online impulsive purchasing (Akram et al., 2018a).

Involvement mediates between perceived e-commerce anchor attributes, perceived scarcity, and immersion and impulsive purchase behavior. Partially mediating effects of involvement are found between perceived e-commerce anchor attributes and consumer impulsive purchase behavior; and between immersion and impulsive purchase behavior. Involvement fully mediates between perceived scarcity and impulsive purchase behavior. The findings indicate that perceived e-commerce anchor attributes and immersion can directly or indirectly affect impulsive purchase behavior through involvement. However, perceived scarcity only influences impulsive purchase behavior through involvement.

### Implications for Theory

Technological innovations in the Internet and information systems seem to have driven changes in concepts and habits of consumption. A major development of online shopping is livestreaming shopping that is centered on consumer experience, and this study provides an understanding of consumer purchase behavior in livestreaming shopping. Consumer decision-making is greatly influenced by stimuli carefully designed by marketers. The study extends behavioral theory in two aspects.

First, this study innovatively combined the SOR theory of environmental psychology with the “People-Product-Place” marketing model and established a research framework of “marketing method stimulus-involvement-impulsive purchase behavior” corresponding to the three elements of “People-Product-Place.” The study investigated the influencing mechanisms of perceived e-commerce anchor attributes, perceived scarcity, and immersion on impulsive purchase behavior from the consumers’ perspective. The study enriches the theoretical understanding of impulsive purchase behavior in livestreaming shopping and provides a theoretical basis for further research.

Second, previous studies have mostly considered immersion as a mediating factor. This study considered consumers’ perceptions of immersion in livestreaming shopping rooms as an independent variable, expanding the research scope of immersion.

### Implications for E-commerce and Its Regulation

Path analysis indicates that immersion is the strongest predictor of involvement and impulsive purchase behavior. Involvement is a significant predictor of impulsive purchase behavior. Thus, anchors, merchants, and platforms should actively expand shopping scenarios, enrich consumers’ experience of watching livestreaming, and fulfill their diverse consumption needs. The consumer experience can be enhanced or optimized using virtual reality, artificial intelligence and big data analytics, enabling consumers to experience immersive shopping, enhancing their sense of authenticity and their trust in online shopping. Marketers can focus on creating a joyful atmosphere in livestreaming shopping rooms so as to infect consumers with the ambience when they watch and experience immersive shopping. Anchors should avoid applying the same marketing strategy to all products but should use different strategies to keep consumers feeling fresh, integrate themselves into the shopping atmosphere, and increase their involvement, and eventually purchase. Perceived scarcity is also a significant predictor of involvement and impulsive purchase. In practice, creating a limited-time-quantity promotion atmosphere is key. Consumers are led by the idea of “what is scarce is valuable.” Perceived scarcity increases perceived value, increases the desire to buy, and enables the goal of product promotion to be achieved.

In China, rapid development and tax evasion by several e-commerce livestreaming shopping anchors have recently brought stricter regulatory standards to the industry, and thus higher implications for its managers. It seems essential for government to strengthen regulation of the five main participants in the livestreaming shopping industry: merchants, anchors, platform operators, anchor service agencies, and consumers. Regulation needs to clarify the rights and responsibilities of these parties and standardize online livestreaming marketing. Relevant regulatory legislation for the livestreaming shopping industry to improve supervision is expected in the future. For platforms, it is necessary to strictly review the qualifications of merchants and anchors, increase the legal awareness and integrity of anchors, and improve their professionalism. The accounts of anchors who deceive and mislead consumers should be banned or closed in a timely manner. The platform should also strictly monitor for livestreaming data fraud, establish an anti-false data supervision mechanism, and purify the e-commerce livestreaming ecosystem. Anchors must promote products according to objective facts; not fabricate or exaggerate effects or facts. As influential public figures, anchors must abide by the law and regulations, be socially responsible, and accept public oversight. Anchors should strive to improve their professionalism by improving product selection standards and product quality, so as to guarantee consumer satisfaction. It is necessary for e-commerce anchors to strengthen their livestreaming image. The greater the charm of the anchor, the more psychological pleasure it can bring to consumers. Anchors should pay attention to the creation of personal image by strengthening their characteristics, creating more interaction with consumers and increasing their stickiness, establishing an emotional connection with fans, and creating a warm and charming anchor image.

China’s livestreaming shopping industry has made remarkable achievements, especially in assisting economic recovery in the current postepidemic period. The success of this industry stems from the reshaping of the established “People-Product-Place” retailing strategy. Hopefully this experience may provide valuable lessons for the livestreaming industry in other countries.

## Study Limitations and Further Research

There are three methodological limitations. One is that respondents are from China. This limitation does not restrict application of the study findings to livestreaming shopping platforms in China. Consumers are stimulated by the “People-Product-Place” marketing strategy. Impulse buying in livestreaming shopping rooms is significantly affected by perceived e-commerce anchor attributes, perceived scarcity, immersion, and involvement. The second methodological limitation is the use of convenience sampling, and more systematic sampling should be used in future studies. Despite these limitations, this study contributes to the literature on impulsive purchase behavior in livestreaming shopping platforms. Thirdly, this study used self-reported data that might not reflect actual decision-making. For instance, consumers may not willingly admit to being impulsive. Further studies could use quasi-experimental methods or a data analytics approach using data provided by shopping platforms.

Further research should also consider different types of e-commerce anchor, including key opinion leaders, celebrities, famous e-commerce anchors, and merchant-employed anchors. These types of anchors might employ different marketing strategies and factors that influence impulsive purchase might differ. Thus, it is recommended to investigate how different factors influence consumer purchase behavior from different types of e-commerce anchors and the specific strategies they apply. Finally, this study selected perceived anchor attributes, perceived scarcity, immersion, and involvement as predictors of impulsive purchase behavior. Further exploration is needed to identify and characterize other influencing factors for impulsive buying and extends the theoretical framework.

## Data Availability Statement

The raw data supporting the conclusions of this article will be made available by the authors, without undue reservation.

## Author Contributions

BC and JW: conceptualization, methodology, software, formal analysis, resources, data curation, writing—original draft preparation, visualization, and project administration. LW and HR: validation, writing—review and editing, and supervision. LW: investigation. All authors have read and agreed to the published version of the manuscript.

## Conflict of Interest

The authors declare that the research was conducted in the absence of any commercial or financial relationships that could be construed as a potential conflict of interest.

## Publisher’s Note

All claims expressed in this article are solely those of the authors and do not necessarily represent those of their affiliated organizations, or those of the publisher, the editors and the reviewers. Any product that may be evaluated in this article, or claim that may be made by its manufacturer, is not guaranteed or endorsed by the publisher.

## Footnotes

1 Stockholm-based live video services providers.

2
https://www.wjx.cn/
